# Associations between individual and structural level discrimination and psychological and physiological stress indicators during pregnancy

**DOI:** 10.1038/s44294-025-00100-z

**Published:** 2025-09-22

**Authors:** Özlü Aran, Melissa Nevarez-Brewster, Kimberly D’Anna-Hernandez, Julia Dmitrieva, Curt A. Sandman, Laura M. Glynn, Elysia Poggi Davis

**Affiliations:** 1 Department of Psychology, University of Denver, Denver, CO USA; 2 Department of Psychology, Northeastern University, Boston, MA USA; 3 Department of Psychology, Marquette University, Milwaukee, WI USA; 4 Department of Psychiatry and Human Behavior, University of California-Irvine, Irvine, CA USA; 5 Department of Psychology, Chapman University, Orange, CA USA; 6 Department of Pediatrics, University of California-Irvine, Irvine, CA USA

**Keywords:** Health services, Physiology

## Abstract

In the United States (U.S.) pervasive health disparities in prenatal care disproportionately impact marginalized individuals. The present study investigates whether discrimination at the individual and structural levels are associated with indicators of psychological and physiological stress among pregnant Latinx people living in the U.S., including both U.S. and foreign-born individuals. Pregnant participants (*n* = 109) reported on birthplace, lifetime experiences of racial and ethnic discrimination, perceived levels of stress, acculturation, social support, and provided residential address data and hair samples for cortisol analysis. Regression analysis revealed that individual level discrimination was linked to higher psychological stress during pregnancy (*p* = .003), and structural level discrimination was related to lower physiological stress (cortisol; *p* = .056). Notably, these associations varied by birthplace: U.S.-born individuals showed higher levels of psychological stress in response to individual level discrimination, while foreign-born individuals appeared more resilient to structural level discrimination. The results are discussed within the framework of immigrant health paradox.

## Introduction

Health disparities in the United States (U.S.) are pervasive and disparities in pregnancy complications and birth outcomes are particularly critical^1,2^. Rates of prenatal maternal morbidity and mortality as well as preterm birth are exceptionally high among individuals from marginalized backgrounds^3^ (https://stacks.cdc.gov/view/cdc/124678). The fact that 84% of pregnancy-related deaths are preventable in the U.S^4^. demonstrates the urgent need to identify the mechanisms through which inequities contribute to prenatal health disparities. The Latinx population needs a special attention in this regard as the fastest growing group with the highest birth rates (https://www.census.gov/newsroom/press-releases/2024/population-estimates-characteristics.html) in the U.S. (The authors acknowledge that the categorizations of race and ethnicity are social constructs and further there is not a universal consensus on the preferred terminology. Latinx is a gender-neutral, umbrella term used to identify the ethnicity of individuals who originate from Central and South America, Caribbean countries, and Mexico (https://apastyle.apa.orghttps://apastyle.apa.org/style-grammar-guidelines/bias-free-language/racial-ethnic-minorities). Therefore, we used the terms “Latinx” and “pregnant individuals” to refer to the participants of the current study).

Discrimination affects the health of marginalized populations at multiple levels^5–7^ including racial and ethnic discrimination at the individual and structural levels^8,9^. Individual level discrimination includes day-to-day interactions with people on the street, at the workplace, or in community settings. Structural level discrimination includes management, maintenance, and enforcement of racial and ethnic discrimination and inequities through political and societal determination of access to and allocation of resources and opportunities in neighborhoods such as access to education, healthcare and opportunities for housing and employment^10^. Discriminatory policies and practices lead to structural inequities in various levels of life including within neighborhoods. The Integrative Model^8^ and the Phenomenological Variant of Ecological Systems Theory (PVEST)^9^ state that interactions within the family or with peers and the neighborhood can impact development through racism, discrimination and environmental segregation experienced in school systems, social resources, and health care. Stratification of families into lower socioeconomic environments with fewer resources are dynamically shaped by and interact with political, cultural, social values and ideologies and exert a risk for racially and ethnically minoritized children’s development. As such, experiences of racial and ethnic discrimination can influence both psychological and physiological stress systems during pregnancy and are major risk factors for racially and ethnically marginalized communities including Latinx population^11^. The current study focuses on pregnancy, a sensitive period during which environmental inputs can alter development and impact the health of the pregnant parent and the fetus^12–14^. Stress responses during pregnancy are particularly important because they program fetal development and can affect birth outcomes^15,16^. We investigate the links between discrimination at the individual and structural levels of environmental stressors and indicators of psychological and physiological stress among Latinx pregnant individuals.

Experiences of racial and ethnic discrimination at the individual level impact mental health during pregnancy. Both in general population and among the Latinx, individual level discrimination is associated with higher prenatal depression^17–21^ and anxiety symptoms^19,22^. Discrimination at the individual level additionally presages higher psychological stress during pregnancy^22–24^. Fewer studies have evaluated links between discrimination and physiological stress indicators during pregnancy. The hypothalamic-pituitary-adrenal (HPA) axis regulates the physiological stress response system and the production of the stress-responsive hormone, cortisol (see^25,26^ for reviews). Cortisol plays a multitude of roles in the body including regulation of stress responses, inflammation and metabolism. During pregnancy, cortisol plays a key role in regulation of fetal maturation^27^. Thus, alterations of the HPA axis functioning during pregnancy is a plausible process that plays a key role in the intergenerational transmission of health disparities and studies investigate cortisol as an end product of the HPA axis^28,29^. Racial and ethnic differences in cortisol levels and profiles among individuals from marginalized backgrounds are likely explained by exposure to racial and ethnic discriminatory practices^30–32^. For example, more experiences of discrimination are associated with a blunted cortisol response to acute stress^33^ and higher cortisol levels in the evening^34^. To our knowledge, there is not a study investigating the effects of discrimination on HPA axis functioning among pregnant Latinx individuals. Further, research is needed to understand how experiences of discrimination related to chronic stress exposure to the HPA axis. Cortisol measured in hair, as a novel approach to assess prenatal stress physiology, allows for assessment of chronic stress and can provide information on the stress levels of the individual during the pregnancy period.

Discrimination at the structural level is another environmental stressor that impacts prenatal health. Living in neighborhoods with fewer resources is associated with pregnancy-related complications^35–37^ and preterm birth^38,39^. Studies assessing neighborhood structural inequities have historically included only one or two neighborhood indicators, such as neighborhood poverty or safety^40–42^. However, discrimination at the structural level can be experienced in many forms including but not limited to educational and housing access. Spatial data science provides a new approach to examining the health impacts of access to and allocation of resources in neighborhoods^43^. The current study utilizes a neighborhood adversity metric, the Area Deprivation Index (ADI), characterizing neighborhood features such as educational level among residents, income disparity, housing market, and household characteristics using a composite score^44^ (https://www.neighborhoodatlas.medicine.wisc.edu). Higher ADI presages poorer medical outcomes during pregnancy including higher rates of infectious disease, mortality and morbidity as well as increased inflammation^45–49^, indicating that structural discrimination likely affects prenatal health. Consistent with this hypothesis one study in Montreal, Canada finds greater depression symptoms and stress among pregnant individuals living in neighborhoods with higher neighborhood deprivation index^50^. Our understanding of the association between neighborhood deprivation and stress outcomes during pregnancy in the U.S. remains unknown. The percentage of Latinx individuals living in segregated and underresourced neighborhoods compared to non-Latinx White people is higher in the U.S^51^ and neighborhood deprivation is related to high stress and poor metabolic health among non-pregnant Mexican Americans, the largest Latinx subgroup in the U.S^52,53^. As such, studies examining specifically pregnant Latinx population are needed.

The sociocultural climate of the U.S. directly affects the health of racially and ethnically marginalized people^54,55^ (https://www.cdc.gov/nchs/data/factsheets/factsheet_disparities.htm). Accumulating research shows that while U.S.-born Latinx individuals have poor health compared to non-Latinx White Americans, their foreign-born counterparts have similar health quality to non-Latinx White Americans even when they have lower socioeconomic status^56,57^. Similarly, foreign-born individuals from various countries who reside in the U.S. show a decline in physical and mental health as duration of stay in the U.S. increases^58,59^ and the negative impact of the environment on health persists in the subsequent generations^60^. This pattern, often referred to as the *immigrant health paradox*, has been consistently documented within many immigrant populations including Latinx communities. Pregnancy is a sensitive window during which tremendous physiological and psychological changes occur both for the parent and fetus potentially increasing susceptibility to environmental signals^61^. Indeed, differences in pregnancy outcomes based on the level of exposure to the U.S. culture are documented within *immigrant health paradox* literature. When compared to U.S.-born pregnant individuals, foreign-born individuals residing in the U.S. show fewer pregnancy complications and more optimal birth outcomes^62–64^. This has highly important implications across generations as the health decline from one generation to the next may impact birth outcomes negatively^61^ and thus it highlights the need for considering birthplace to disentangle the experiences of Latinx communities as the largest growing group with high birth rates in the U.S.

Research on Latinx populations identifies protective factors such as diet and immigrant enclaves in neighborhoods which were proposed as benefits contributing to the *immigrant health paradox*^65,66^. Acculturation and social support are two additional factors that may contribute to the *immigrant health paradox* based on the premise that Latinx individuals who moved to the U.S. are more likely to maintain their cultural values and social ties with their community relative to U.S.-born Latinx individuals. Low levels of acculturation and high levels of social support may serve as protective factors against the potential health impacts of adversities in an oppressive system. For example, acculturation measured as more years in the U.S. predicted worse health behaviors among a Mexican American sample of pregnant individuals. However, high social support attenuated this association and was associated with better health behaviors^67^. Similarly, studies with Latinx pregnant individuals show the protective role of social support on birth outcomes^68,69^. Hence, in the current study we implement exploratory analyses examining birthplace of individuals (U.S. or Latin America) as a way of testing whether the *immigrant health paradox* extends to prenatal psychological and physiological stress. Further, we test whether acculturation and social support explain the associations between individual and structural level discrimination and psychological stress and hair cortisol during pregnancy.

Few studies have evaluated the effects of individual and structural racial/ethnic discrimination on psychological and physiological stress during pregnancy. This study aims to investigate how discrimination at different levels contribute to psychological and physiological stress during pregnancy among Latinx pregnant individuals living in the U.S., 55% of whom were foreign-born. We examined racial and ethnic discrimination at the individual and structural levels to expand our current understanding on prenatal health disparities. We hypothesized that discrimination at individual and structural levels would be associated with psychological stress during pregnancy and prenatal hair cortisol levels among Latinx pregnant individuals. Further, we included exploratory analyses with birthplace, acculturation and social support as moderators that could explain the associations between discrimination and psychobiological stress during pregnancy.

## Results

### Descriptive statistics

Preliminary analysis examining the distributional properties of and bivariate correlations among the study variables were performed. Table 1 summarizes descriptive statistics for study variables in the total sample and for the U.S.- and foreign-born groups as well as differences between U.S.- and foreign-born groups. See Table 2 for correlations among study variables.

**Table 1:** Descriptive statistics for the total sample, groups and group differences based on birthplace

	Total sample *n* = 109	Foreign-born *n* = 60	U.S.-born *n* = 49	Group Differences (two tailed *t*tests)
**Main Analysis Variables**	***M (SD)***			***t*(df)*, p***
Psychological stress during pregnancy	13.9 (6.6); 1–37	14.3 (6.5)	13.4 (6.7)	*t*(107) = 0.743, *p* = 0.459
Prenatal hair cortisol (pg/mg)	9.0 (13.1);.9-115.9	8.7 (6.7)	9.3 (18.1)	*t*(85) = 1.211, *p* = 0.229
Experiences of discrimination	1.3 (1.8); 0–9	1.2 (1.4)	1.5 (2.1)	*t*(81.0) = –0.680, *p* = 0.498
Area Deprivation Index	23.6 (20.3); 2–100	25.3 (21.3)	21.6 (19.0)	*t*(107) = 0.827, *p* = 0.410
**Moderators & Covariates**	***M (SD)***			***t*(df)*, p***
Social support	83.8 (17.2), 31.6–100	78.4 (17.4)	90.4 (14.6)	*t*(107) = –3.908, *p* < 0.001
Acculturation	–0.3 (2.1); -4–4	-1.4 (1.7)	1.1 (1.6)	*t*(107) = –7.653, *p* < 0.001
GA at assessment (weeks)	28.9 (6.5); 18.5–46.8	28.8 (3.3)	28.2 (3.2)	*t*(107) = 0.981, *p* = 0.329
Age at delivery (years)	28.9 (6.5); 18.5–46.8	31.1 (6.4)	26.2 (5.8)	*t*(107) = 4.145, *p* < 0.001
Years of education	12.4 (2.5); 2–17	11.4 (2.8)	13.6 (1.3)	*t*(85.8) = –5.215, *p* < 0.001
Income-to-needs ratio	177 (197.3); 0–1,227	130.7 (108.4)	233.8 (259.2)	*t*(61.7) = –2.605, *p* = 0.011

For hair cortisol and Area Deprivation Index raw values were reported for means and standard deviations (SDs) and log10 transformed variables were used in analyses. Hair cortisol *n* = 87 (47 foreign-born, 40 U.S. born).

*GA* gestational age.

**Table 2:** Correlations among study variables

	1	2	3	4	5	6	7	8	9
1. Psychological stress during pregnancy									
2. Prenatal hair cortisol (log10)	**0.20^**								
3. Experiences of discrimination	0.30**	**0.07**							
4. Area Deprivation Index (log10)	**–0.09**	**–0.20^**	0.01						
5. Acculturation	**0.02**	**–0.02**	0.13	–0.17^					
6. Social support	–0.30**	**–0.16**	–0.07	0.02	0.20*				
7. Gestational age at assessment	–0.02	0.19^	–0.08	–0.02	–0.04	–0.07			
8. Age at delivery	**–0.03**	**–0.01**	0.13	0.04	–0.23*	–0.08	0.12		
9. Years of education	**0.01**	**–0.04**	0.12	–0.15	0.63***	0.21*	0.07	–0.27**	
10. Income-to-needs ratio	**–0.09**	**–0.08**	–0.09	–0.08	0.35***	0.27**	–0.04	–0.05	0.31**

^*p* < 0.10, *p < 0.05 **p < 0.01, ***p < 0.001. Correlations with psychological stress and prenatal hair cortisol are reported after covarying for gestational age at assessment given the dynamic changes over pregnancy. These correlations were presented as bolded. All other correlations are bivariate correlations.

### Do discrimination at individual and structural levels relate to stress indicators?

**Fig. 1:**
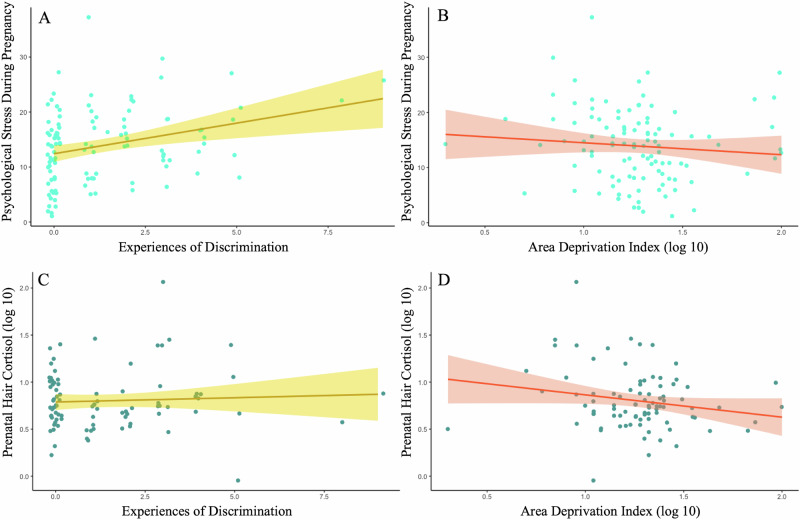
Associations between individual and structural discrimination and the psychological and physiological stress indicators. Model **A**: Individual level discrimination & psychological stress during pregnancy. Model **B**: Structural level discrimination &psychological stress during pregnancy. Model **C**: Individual level discrimination & physiological stress during pregnancy. Model **D**:Structural level discrimination & physiological stress during pregnancy. All models covaried for gestational age at assessment,parental age at delivery and years of education

Linear regression models revealed that participants reporting greater experiences of discrimination had higher levels of psychological stress during pregnancy (*B* = 1.112, *SE* = 0.362, *p* = .003; Fig. 1A, Table 3 Model 1, Table S2 in Supplementary material). Experiences of discrimination were not significantly related to prenatal hair cortisol levels (*B* = 0.016, *SE* = 0.019, *p* = 0.406; Fig. 1C, Table 3 Model 1, Table S2). Area deprivation was not significantly associated with psychological stress during pregnancy (*B* = –2.259, *SE* = 2.293, *p* = 0.327; Fig. 1B, Table 3 Model 2), but was associated with lower physiological stress as indicated by prenatal hair cortisol levels (*B* = 0.255, *SE* = 0.131, *p* = .056; Fig. 1D, Table 3 Model 2), although the association with area deprivation did not reach the traditional thresholds of significance.

**Table 3:** Regression models of experiences of discrimination, area deprivation, and the indicators of psychological and physiological stress during pregnancy

		Psychological Stress During pregnancy					Prenatal hair cortisol				
Model	Predictors	*B*	*SE*	β	*t*	*p*	*B*	*SE*	β	*t*	*p*
**1**	Experiences of discrimination	1.112	0.362	0.301	3.007	0.003**	0.016	0.019	0.095	0.836	0.406
	Gestational age at assessment	0.008	0.194	0.004	0.043	0.966	0.020	0.011	0.209	1.790	0.077^
	Age at delivery	–0.025	0.101	–0.025	–0.250	0.803	–0.001	0.006	–0.021	–0.174	0.863
	Years of education	–0.037	0.280	–0.014	–0.133	0.894	–0.006	0.015	–0.051	–0.396	0.693
	Income-to-needs ratio	–0.002	0.003	–0.056	–0.563	0.574	–0.000	0.000	–0.021	–0.177	0.860
**2**	Area Deprivation Index	–2.259	2.293	–0.097	–0.985	0.327	–0.255	0.131	–0.209	–1.930	0.056^
	Gestational age at assessment	–0.071	0.200	–0.035	–0.357	0.722	0.018	0.011	0.188	1.682	0.096^
	Age at delivery	0.037	0.103	0.037	0.362	0.718	0.000	0.006	0.007	–0.370	0.712
	Years of education	–0.116	0.287	0.044	–0.404	0.687	–0.007	0.013	–0.045	–0.552	0.583
	Income-to-needs ratio	–0.004	0.003	–0.109	–1.056	293	0.000	0.000	–0.048	–0.409	0.683

^*p* < 0.10, *p < 0.05 **p < 0.01, ***p < 0.001.

### Do the associations between discrimination and stress indicators differ by birthplace?

**Fig. 2:**
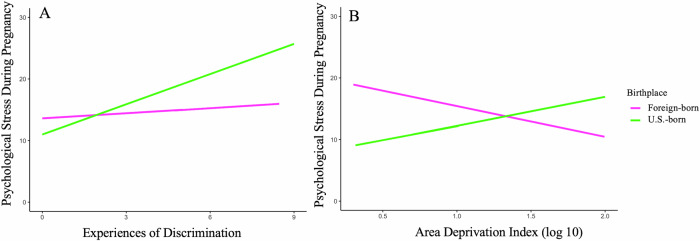
Moderating role of birthplace in the link between discrimination and the psychological stress indicator. Model **A** Individual level discrimination & psychological stress moderated by birthplace. Model **B**: Structural level discrimination & psychological stress moderated by birthplace.

To probe interactions between birthplace and discrimination on stress outcomes, we explored participant birthplace as a moderator. Birthplace moderated the associations between both individual and structural discrimination and psychological stress during pregnancy (Interaction between birthplace and experiences of discrimination *B* = 1.401, *SE* = 0.707, *p* = 0.049; Fig. 2A, and interaction between birthplace and area deprivation: *B* = 10.609, *SE* = 4.675, *p* = 0.025; Fig. 2B). Overall, interactions revealed that the association between experiences of discrimination and psychological stress differed for the U.S.-born vs. foreign-born individuals (See Fig. 2). Specifically, simple slope analysis revealed that greater experiences of discrimination were associated with higher levels of psychological stress during pregnancy among the U.S.-born (*B* = 1.640, *SE* = 0.420, *p* < 0.001) but not foreign-born individuals (*B* = 0.230, *SE* = 0.570, *p* = 0.680; Fig. 2A). For area deprivation, higher deprivation was associated with lower psychological stress during pregnancy among the foreign-born (*B* = –5.950, *SE* = 2.730, *p* = .030) but not U.S.-born individuals (*B* = 4.660, *SE* = 3.790, *p* = 0.220; Fig. 2B). Birthplace did not significantly moderate the associations between experiences of discrimination or area deprivation and prenatal hair cortisol (Interaction between birthplace and experiences of discrimination: *B* = –0.025, *SE* = 0.039, *p* = 0.518; and interaction between birthplace and area deprivation: *B* = –0.081, *SE* = 0.268, *p* = 0.764).

### Do the associations between discrimination and stress indicators differ by acculturation and social support?

**Fig. 3:**
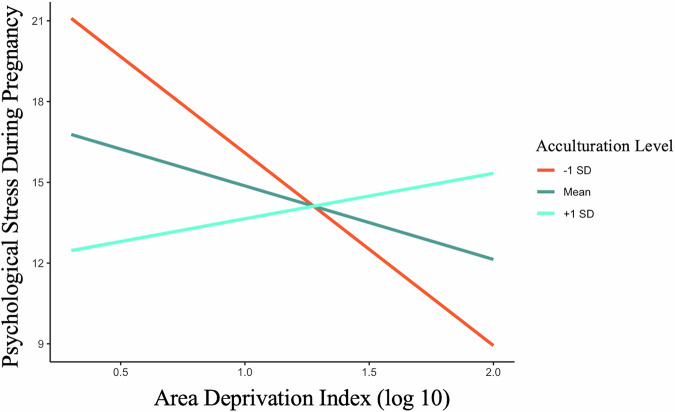
Moderating role of acculturation in the link between structural discrimination and the psychological stress indicator. Those with acculturation levels that are 1 standard deviation (-1 SD) below the mean reported less stress when they lived in more deprived neighborhoods.

To further understand the different patterns in associations between experiences of discrimination, area deprivation, and psychological stress during pregnancy shown by the U.S.- and foreign-born participants, we explored acculturation and social support as potential moderators. As expected, acculturation was higher among the U.S.-born compared to the foreign-born individuals (See Table 1). However, acculturation did not significantly moderate the association between experiences of discrimination and psychological stress during pregnancy (Interaction between acculturation and experiences of discrimination: *B* = 0.146, *SE* = 0.173, *p* = 0.398). Acculturation moderated the association between area deprivation and psychological stress (Interaction between acculturation and area deprivation: *B* = 2.129, *SE* = 1.117, *p* = 0.060; Fig. 3), although this association did not reach the traditional thresholds of significance. Simple slope analysis revealed that those who had the lowest acculturation levels reported less stress when they lived in more deprived neighborhoods (*B* = -7.170, *SE* = 3.460, *p* = 0.040, Fig. 3). Social support was also higher among the U.S.-born relative to the foreign-born individuals (See Table 1), however, social support did not significantly moderate associations between experiences of discrimination (Interaction between social support and experiences of discrimination: *B* = 0.015, *SE* = 0.018. *p* = 0.413) or area deprivation and psychological stress during pregnancy (Interaction between social support and area deprivation: *B* = 0.074, *SE* = 0.139, *p* = 0.597).

## Discussion

Disparities within the U.S. have profound implications for the health and development of pregnant individuals. Individual and structural level discrimination were associated with different stress indicators during pregnancy among Latinx individuals. Discrimination at the individual level presaged higher reports of psychological stress whereas structural level discrimination was linked to lower hair cortisol levels (physiological stress indicator) although the latter association did not reach the traditional thresholds of significance. Notably, the pattern of these associations differed by birthplace. Structural and individual level experiences of discrimination had negative implications for psychological stress among the U.S.-born individuals whereas foreign-born individuals were relatively protected. Consistent with *the immigrant health paradox*, the present findings suggest that the U.S.-born Latinx pregnant individuals might be impacted more negatively from the sociocultural climate of the U.S. than foreign-born individuals.

Exposure to individual level discrimination was associated with higher psychological stress during pregnancy. This finding is consistent with the previous work showing that experiences of discrimination presage worse mental health, higher psychological stress, and poorer birth outcomes among pregnant individuals^17,20,22,69^. Structural discrimination on the other hand, was modestly associated with the physiological (*p* = 0.056), but not psychological stress indicator. Finding that individual and structural discrimination differentially relate to indicators of psychological and physiological stress during pregnancy is consistent with previous work demonstrating an uncoupling between psychological and physiological indicators of stress^70,71^. Further, results are similar to other studies in pregnancy which do not detect associations between measures of psychological stress and cortisol^72,73^. It is plausible that individual level discrimination relates more strongly to the psychological stress indicator because both reflect individual perceptions of experience. It is unclear why structural level discrimination associates more strongly with physiological stress. Stress physiology is dynamic across pregnancy and thus it is plausible that ongoing exposures to neighborhood factors impact prenatal physiology during this dynamic period. Future work investigating additional measures of physiological stress (e.g. placental corticotropin-releasing hormone) and psychological stress are needed to further elucidate the differential impact of individual and structural level discrimination.

Consistent with the *immigrant health paradox*, implications of individual and structural discrimination on psychological stress differed based on birthplace. Specifically, foreign-born individuals were less susceptible to discrimination experiences. Psychological stress during pregnancy was higher among U.S.-born participants with greater exposure to individual level discrimination whereas foreign-born individuals showed lower psychological stress pattern with higher structural discrimination. It is proposed that foreign-born individuals who move to the U.S. may experience fewer health consequences related to discrimination^66^. Increased duration of residence in the U.S. and exposure to discrimination in this new context may lead to deterioration in health^66,74^. Despite the consistent findings on the immigrant health paradox among the Latinx, there are alternative explanations for the paradox due to high variability in findings based on race, ethnicity, and geographic location^51^. Although not in the scope of the current study to test, these alternative explanations are important to acknowledge. Sociological explanations for the paradox state that the reason for paradox could be migrant selectivity theory (more healthy individuals immigrate to the U.S.) or salmon bias hypothesis (individuals move back to their home countries following deterioration in health)^75^. Methodological alternative explanation links the paradox findings to data bias and artifacts such as lower rates of health reporting among immigrants, underreporting of death certificates, biases in the definition of Latinx construct (see^74^ for a review).

Lower levels of acculturation were hypothesized as one factor contributing to resilience to interpersonal signs of discrimination among foreign-born individuals^66^. Consistent with this hypothesis, in the current study, we found that the foreign-born group showed lower acculturation levels compared to the U.S.-born group. However, we did not have the statistical power to test whether acculturation explained the associations between discrimination, birthplace, and stress indicators. One speculation for lower psychological stress with higher ADI among the foreign-born group is the potential protective factors in neighborhoods with high deprivation. It is the case that living in immigrant enclaves protects against various health outcomes among foreign-born Latinx individuals^76,77^ and higher Latinx concentration is related to lower neighborhood income but also lower postpartum depression symptoms^78^. Thus, it is plausible that individuals living in neighborhoods rated as more deprived based on the ADI also experience more inclusive and supportive living environments. The ADI focuses on the structural issues such as income and employment, and housing market and household characteristics. These U.S.-specific measures do not address the entire context of foreign-born individuals or how they perceive the resources in the neighborhoods they reside. For example, women who live in immigrant enclaves and report lower acculturation have lower levels of depression symptoms^79^. Consistent with this, we found that lower acculturation was protective against the effect of high structural discrimination on psychological stress during pregnancy. Similarly, one study documented lower prenatal depression and anxiety among Latinx pregnant individuals living in highly segregated neighborhoods^80^ and another study showed an association between residential segregation and poor pregnancy outcomes only among U.S.-born but not foreign-born Black pregnant individuals^81^. Alternatively, prior findings show that both low and high cortisol levels are predictive of poor health^73,82^. For example, early trauma such as maltreatment or abuse during childhood is associated with lower baseline cortisol levels^83^. As such, lower hair cortisol levels among the foreign-born group could potentially reflect a negative outcome.

Social support, another plausible protective factor, was higher among U.S.-born individuals but did not buffer against the effects of discrimination in the current study. It could be that social support could not buffer against the detrimental effects of discrimination. However, findings in the existing literature are conflicting about the buffering role of social support among Latinx and immigrant populations^69,84,85^, possibly due to variations in how social support is measured within this literature. The types and aspects of social support as well as who provides the support could matter and the measure included in our study was a composite score that did not evaluate support types, aspects or providers. There is evidence that other components of social support such as the number of individuals that a person receives social support from may differ between U.S.- and foreign-born individuals^85^. Further, social support is a multilayered construct and other protective factors in the Latinx context such as *familismo* (familism) are important to consider^84,86^. Lack of a comprehensive social support measure limits the ability to identify the role of social support in the current study.

The current study should be evaluated in the context of its strengths and limitations. A major strength of this study is the inclusion of Latinx pregnant individuals, allowing for the examination of the unique experiences of this population. Although all participants lived in the same region in the U.S., a limitation of this study was grouping those who were born in different parts of Latin America (e.g., Mexico, Guatemala) into one foreign-born group. Given the enormous range in experiences and rich cultures of persons with Latinx heritage, there are multiple cultural and contextual factors beyond nationality to consider such as specific racial and ethnic identification. Differences in country of origin as well as generational status could also inform differences in health status when individuals arrive in the U.S.^60,87^. Future studies should take these nuances into account to avoid generalizing experiences across different ethnic groups. Another strength of the current study is the inclusion of both psychological and physiological indicators of stress. Psychological and physiological stress processes are interactive^25^ and investigating indicators of both psychological and physiological stress allows for a comprehensive understanding of the effects of discrimination on stress. However, the assessment of stress indicators only at one time point with a relatively small sample size limited the current study to test these possibilities. Future longitudinal studies with larger samples should address these limitations and extend the present findings by conducting mediation analyses with psychological stress indicators predicting physiological stress. Finally, this study included both individual and structural levels of discrimination. These novel findings support previous work on experiences of discrimination and leverage the ADI as a proxy of structural discrimination. Of note, almost half of the sample did not report any experiences of discrimination. This is consistent with previous literature with pregnant as well as Latinx samples^20,88,89^. Future studies could aim to understand this observation and further probe the extent to which it is due to measurement limitations and perceived experiences. We demonstrate that the ADI can provide additional information to reveal the complexities of discrimination and how they manifest in stress outcomes. However, the sample comprises individuals living in southern California and the data collection was between 2011 and 2015. This limits generalizability to other regions of the U.S. and may not reflect the current stress experiences in the U.S. Changes in the sociopolitical climate with time can inform parental and offspring health^90^ and these nuances should be considered in the interpretation of the results. Information on how long participants lived at their addresses and moving history were not assessed. Future studies including geocoding data should gather more detailed address history from participants to overcome these limitations and test these associations with other neighborhood indices including metrics such as healthcare and educational resources, environmental pollutants, and green space. Further, future research should consider cumulative and synergistic relations between individual and structural levels of discrimination on prenatal stress indicators. Finally, although we explore the moderating effects of birthplace, acculturation, and social support, there may be other factors buffering the effects of discrimination on stress, including perceived social status. Future studies should test additional factors, such as perceived social status, as buffers linking multilevel discrimination and stress during pregnancy^91^.

Overall, the current study highlights the differential associations among discrimination at the individual and structural level, and indicators of psychological and physiological stress during pregnancy. Findings were consistent with the *immigrant health paradox*, suggesting that within the sociocultural climate of the U.S., U.S.-born pregnant Latinx individuals have poorer stress outcomes compared to foreign-born pregnant Latinx individuals. Prenatal health disparities are pervasive in the U.S. and the current study provides further understanding of how discrimination can impact stress processes among Latinx individuals as the largest growing group of immigrants with high birth rates. Stress during pregnancy is related to a higher number of pregnancy and obstetric complications as well as poor birth outcomes. Hence, understanding the experiences of Latinx pregnant individuals and risk factors specific to this group will help ultimately create culturally sensitive interventions that seek to improve parental and offspring health.

## Methods

### Participants and procedure

Participants included 109 Latinx pregnant individuals from a longitudinal study that recruited a pregnancy cohort at the University of California, Irvine Medical Center between 2011 and 2015. Procedures were approved by the Institutional Review Boards of the University of California, Irvine (2010-7592, 2011-8346), Chapman University (1213H079), and the University of Denver (2012-2375). All participants gave written and informed consent, and the study has been performed in accordance with the Declaration of Helsinki. The longitudinal study oversampled pregnant people identified by their attending obstetrician as in need of medical care and at high risk of preterm birth. The current study was a secondary data analysis with the Latinx subset of the sample. Inclusion criteria for the current study included (a) 18 years of age and older, and (b) absence of an endocrine disorder (e.g., any disorder that causes disruption to the endocrine system including hyperthyroidism and Cushing’s disease), prenatal corticosteroid exposure prior to recruitment (e.g., administration of dexamethasone or betamethasone), in vitro fertilization, or major fetal or chromosomal anomalies. Exclusion criteria included (a) regular tobacco use, (b) having more than 5 alcoholic drinks, and (c) any illicit drug use during pregnancy.

Between 23 and 34 gestational weeks, the participants completed the study questionnaires and provided hair samples and their street addresses. More than half of the participants were born outside of the U.S. (*n* = 55 Mexico, *n* = 1 Guatemala, *n* = 1 El Salvador, *n* = 2 other Latin American country) and had lived in the U.S. an average of 15.4 years (*SD* = 7.3, Range = 1.3–36). Nearly 80% of the participants lived below 200% of the federal poverty line and 70% had attained a high school degree or less. See Table 4 for participant characteristics.

**Table 4:** Sample characteristics

Demographics	*M (SD);* Range or % *n*
Income-to-needs ratio (median)	137 (197.3); 0–1227
<100% Federal poverty line	42.2%
100-200% Federal poverty line	37.6%
>200% Federal poverty line	20.2%
Education (highest degree obtained)	
Less than high school degree	37.1%
High school, GED, technical or vocational school	33.3%
College without a degree	16.7%
Associate degree or Certificate	9.2%
Bachelor’s degree	3.7%
Age at delivery	28.9 (6.5); 18.5–46.8
Cohabitating with fetus’ biological father	80.6%
Parity	
No live birth prior current pregnancy	35.2%
One live birth prior current pregnancy	28.7%
Two+ live births prior current pregnancy	36.1%
Birthplace	
Foreign-born	55%
U.S.-born	45%
Primary language	
Spanish	58.9%
English	41.1%

### Individual level discrimination measure

The Experiences of Discrimination Scale (EOD)^92^ was used to assess racial and ethnic discrimination at the individual level. The EOD assesses lifetime racial and ethnic discrimination experienced in nine settings such as, workplace, doctor’s office, and bank while getting housing bank loans. Participant responses to EOD are *never* (0), *once* (1), *2 or 3 times* (2), and *4 or more times* (3). These responses can be used to calculate a count score of the EOD reflecting whether any experience was reported for each setting (0 vs. 1, 2, or 3). The count score can range from 0 to 9, with higher values indicating more settings in which discrimination was experienced. The EOD possesses good reliability^93^ and construct validity^94^. The EOD is also correlated with psychological and physiological stress measures in non-pregnant Latinx adult populations^93^ and pregnant individuals^20^. Experiences of discrimination variable in the current study was skewed because 48% of the sample did not report any experiences of discrimination. Thus, we also tested experiences of discrimination as a binary predictor (presence of discrimination experience or absence of discrimination experience at any setting) of stress indicators (see Tables S1, S2 in Supplementary material).

### Structural level discrimination measure

The Area Deprivation Index (ADI; https://www.neighborhoodatlas.medicine.wisc.edu)^44^ was used to asses inequities in neighborhoods as a structural level factor where study participants resided during pregnancy. As a measure validated and used in the medical research^95,96^, we selected the ADI to assess structural neighborhood factors that can have an impact on psychological and physiological stress levels of pregnant individuals via deprivation in resource access and allocation. The ADI is publicly available geocoding map data based on census blocks determined by the U.S. Census Bureau. The ADI is a composite score of 17 indicators examining four different domains: *Education* (% population aged 25 years or older with less than 9 years of education, % population aged 25 years or older with at least a high school diploma, and % employed population aged 16 years or older in white-collar occupations), *Income/employment* (median family income in U.S. dollars, income disparity, % families below federal poverty level, % population below 150% of federal poverty level, % civilian labor force population aged 16 years and older who are unemployed), *Housing* (median home value in U.S. dollars, median gross rent in U.S. dollars, median monthly mortgage in U.S. dollars, % owner-occupied housing units, % occupied housing units without complete plumbing), *Household characteristics* (% single-parent households with children younger than 18, % households without a motor vehicle, % households without a telephone, % households with more than 1 person per room). The composite score of the ADI ranges between 1 (*least disadvantaged*) and 10 (*most disadvantaged*) and allows for nation and state-wide comparisons. In the current study national ADI score was used given the broader level of comparison it offers in relation to the state score, and we used ADI version from 2015 to match the data collection timeline. We used nine-digit zip code for each participant to calculate the ADI and screened for clustering. All participants lived in different census blocks; thus, the data did not show any clustering. Due to skewed distribution, the ADI score was log 10 transformed in the current study.

### Psychological stress during pregnancy measure

Psychological stress during pregnancy was assessed with the Perceived Stress Scale (PSS)^97^. The current study included the 10-item version of the PSS, a widely used, valid measure. Four items in the measure are reversed-scored (e.g., “*How often have you felt confident about your ability to handle your personal problems?*”) with responses ranging from *never* (0) to *very often* (4). Thus, a sum score of perceived stress ranges between 0 and 40 with higher values indicating greater perceived stress. Psychometric properties of the PSS are well established^98^ and have been validated across cultures^98–100^. In this study, the internal consistency of the PSS was good with a Cronbach’s α of.80.

### Prenatal hair cortisol measure

Hair samples were collected from the posterior vertex region of the head and as close to the scalp as possible. The hair sample was then placed on top of 100% cotton paper, and the paper was folded around the hair and affixed with paper ties at both ends. Samples were stored in a dry and dark drawer until assayed. The three centimeters closest to the root end of the hair samples were assayed for cortisol concentrations, providing a measure of cumulative cortisol production over the previous three months^101^. Thus, a single hair sample provides an index of chronic cortisol production in contrast to momentary measures such as blood or saliva, which require repeated assessment to estimate chronic cortisol release^102^. Hair cortisol was assayed by an independent laboratory (Behavioral Immunology and Endocrinology Lab)^103^ using enzyme-linked immunosorbent assay (ELISA) kits (Salimetrics, LLC, State College, Pennsylvania). Hair was washed 2–3 times for 3 min each in 2.5 mL isopropanol, then pulverized with a Retsch ball mill at 25 Hz for 10 min. Samples were then extracted using 1 mL of methanol, then left to incubate for 24 h at room temperature. Samples were shaken gently during extraction, then evaporated under a steady stream of nitrogen at 38 °C for 30 min. Solvent was reconstituted using 400 μL of phosphate-buffered saline. Samples were centrifuged at 500 rpm during assay, and cortisol was quantified at wavelength 450 nm. Pooled hair samples from random hair clippings were created, ground, and separately mixed to create a laboratory control. These controls served as an internal assay control that was included in every assay for determination of intraassay coefficient of variabilities (CVs). The mean intra-assay CVs were 3.8% and 8.3% for the low and high controls respectively. Inter-assay CV was 1.8%. The high control was spiked with known amounts of cortisol using Salimetrics standards between 0.11 and 3.00 μg/dl. Recovery for these standards averaged 92.5%. A limiting dilution of the high internal laboratory control from 1:1 to 1:64 showed near perfect linear dilution, R2 = 0.99, between expected and observed levels. There were 87 participants who provided hair, and one participant was excluded due to biologically implausible hair cortisol value (greater than 20,000 pg/mg). Cortisol values were skewed and thus were log 10 transformed. Those without a hair sample did not differ from those with a hair sample in gestational age at assessment, age at delivery, education level, and income-to-needs ratio (*p*s > 0.05). The percentage of foreign-born individuals among those without a hair sample was the same as the whole sample (54%).

### Acculturation measure

Acculturation was measured with the Acculturation Rating Scale for Mexican Americans-II (ARSMA-II)^104^. Although the ARSMA was first developed to focus on Mexican culture specifically, revisions to the ARSMA-II facilitated assessment among other Latinx cultural groups^104^. The ARSMA-II has been widely used with mixed Latinx samples^105,106^ as well as Latina pregnant individuals^107,108^. An abbreviated version of the ARSMA-II with 12 items was used in this study with a 5-point Likert scale ranging between 1 (*not at all*) and 5 (*extremely often or almost always*). Half of these items are related to Latinx orientation (e.g., “*My thinking is done in the Spanish language*”) and the other half represents Anglo orientation (e.g., “*I write letters in English*”). From six items for each orientation, mean scores for Latinx and Anglo subscales are calculated. The acculturation score is then computed by subtracting the Latinx orientation subscale from the Anglo subscale. The acculturation score can range between –4 and +4 with higher values indicating greater acculturation (higher Anglo orientation) and lower values indicating less acculturation (higher Latinx orientation). In the current sample, the Cronbach’s alphas for each subscale were very high (α = 0.92 for Latinx orientation and α = 0.91 for Anglo orientation).

### Social support measure

Social support was measured using the Medical Outcomes Study Social Support Survey (MOS-SS)^109^. The MOS-SS has 18 items under four subscales and an additional item. The scale items are measured on a 5-point Likert scale ranging between 1 (*none of the time*) and 5 (*all of the time*). The questionnaire asks participants to report the availability of various types of support in their lives (“*How often is each of the following kinds of support available to you if you need it?*”). Four subscales and sample items for each are as follows: emotional/informational support (e.g., “Someone who understands your problems”), tangible support (e.g., “*Someone to help you if you were confined to bed*”), affectionate support (e.g., “*Someone who hugs you*”), and positive social interaction (e.g., “*Someone to have a good time with*”). A composite social support scale is calculated by averaging all 19 items. The composite social support score can range between 0 and 100 with higher values indicating greater social support. The MOS-SS is a commonly used measure among Latinx pregnant individuals and has demonstrated high reliability and validity^68,69,110^. In the current sample, the Cronbach’s alpha for the composite score of social support was very high (α = 0.95).

### Covariates

Because both psychological stress and cortisol levels during pregnancy change over gestation^111,112^, it is important to covary for gestational age at assessment. Thus, gestational age at prenatal psychological stress and hair cortisol assessment was included as a covariate in all analyses. Furthermore, previous work demonstrates that age at delivery, levels of income, and education are related to psychological stress and hair cortisol during pregnancy^113,114^. Hence, those three variables were included as additional covariates.

### Power analysis

We estimated the sample size with G*Power, 3.1^115^. Based on the effect sizes for the associations between experiences of discrimination and psychological stress during pregnancy as well as stress-related processes and hair cortisol in the previous literature^11,20,22,23,116^, we estimated a medium effect size (partial R2 = 0.09). Using the “linear multiple regression: fixed model, R2 increase” test from the F tests family in G*Power, we would have 80% power to detect a medium effect of R2 = 0.09 at 0.05 alpha level with 82 participants in the sample. Thus, our sample size both for the psychological stress indicator (*n* = 109) and physiological stress indicator (*n* = 87) had sufficient power to test the study hypotheses.

### Missing data

We had missingness in experiences of discrimination, gestational age, age at delivery, education and acculturation variables. We used multiple imputation to address missing data at random. We used income-to-needs ratio, education, birthplace, gestational age at birth, cohabitation status, pregnancy specific anxiety, prenatal anxiety, parity, obstetric risk, social support, and length of residence in the U.S. as auxiliary variables for the multiple imputation.

### Data analysis

First, bivariate correlations among all study variables and identified covariates were conducted. Then, to test the hypothesis that discrimination at individual and structural levels was associated with psychological stress and hair cortisol levels during pregnancy, we conducted linear regression models covarying for gestational age at assessment, parental age at delivery, and years of education. To test the exploratory hypothesis that associations would differ based on birthplace, we included interactions between birthplace (i.e., U.S.-born or foreign-born) and experiences of discrimination and area deprivation in the models. Further exploratory analyses tested whether acculturation or social support moderated the associations between experiences of discrimination and area deprivation and psychological stress and hair cortisol levels during pregnancy. The interaction analyses included gestational age at assessment as a covariate and continuous variables were mean centered. All analyses were conducted in SPSS (descriptives; IBM Corp., 2023) and R software (main analyses and figures; R Core Team, 2021). The underlying code for this study can be made available upon request from the corresponding author.

## Supplementary information

Supplementary Information

## Data Availability

Data can be made available upon request from the corresponding author.
