# Supplementary material for: Associations between individual and structural level discrimination and psychological and physiological stress indicators during pregnancy
Source: NPJ Womens Health. 2025 Sep 22;3(1):52. doi: 10.1038/s44294-025-00100-z (PMC12454116; doi:10.1038/s44294-025-00100-z)
Supplement: Supplementary file 1 — Supplementary Information [file 44294_2025_100_MOESM1_ESM.pdf]

npj Women's Health
Associations between individual and structural level discrimination and psychological and physiological stress indicators during pregnancy

Supplementary Material

Table S1.

Descriptive Statistics for Binary Experiences of Discrimination Variable and Group Differences

	Total sample	Foreign-born	U.S.-born	Group Differences
	n = 109	*n* = 60	*n* = 49	(two tailed t-tests)
**Main Analysis Variables**		***M (SD); %***		***t*(df), *p***
Experiences of discrimination (binary)	0.5 (0.5); 52%	0.6 (0.5); 55%	0.5 (0.5); 49%	*t*(107) = 0.621, *p* = .536

Table S2.

Regression Models of Binary Experiences of Discrimination, and the Indicators of Psychological and Physiological Stress During Pregnancy

	**Psychological stress during pregnancy**					**Prenatal hair cortisol**				
**Predictors**	***B***	***SE***	**β**	***t***	***p***	***B***	***SE***	**β**	***t***	***p***
Experiences of discrimination (binary)	3.378	1.321	.257	2.556	.012	0.010	0.072	.017	0.145	.885
Gestational age at assessment	0.002	0.196	.001	0.011	.990	0.018	0.011	.192	1.632	.107
Age at delivery	-0.038	0.105	-.037	-0.360	.720	0.000	0.006	-.007	-0.054	.957
Years of education	-0.008	0.283	.003	-0.029	.977	-0.003	0.015	-.028	-0.218	.828
Income-to-needs ratio	-0.002	0.003	-.065	-0.640	.524	-0.000	0.000	-.032	-0.267	.790
